# Weaker connectivity in resting state networks is associated with disinhibited eating in older adults

**DOI:** 10.1038/s41366-021-01056-1

**Published:** 2022-01-11

**Authors:** Anthony Brennan, Lars Marstaller, Hana Burianová, David Benton, Claire J. Hanley, Simon Newstead, Hayley A. Young

**Affiliations:** 1 Swansea University, Wales, SA2 8PP UK; 2 Bournemouth University, Fern Barrow, Poole, BH12 5BB UK

**Keywords:** Feeding behaviour, Cognitive neuroscience

## Abstract

**Background/objectives:**

Obesity affects more than forty percent of adults over the age of sixty. Aberrant eating styles such as disinhibition have been associated with the engagement of brain networks underlying executive functioning, attentional control, and interoception. However, these effects have been exclusively studied in young samples overlooking those most at risk of obesity related harm.

**Methods:**

Here we assessed associations between resting-state functional connectivity and disinhibited eating (using the Three Factor Eating Questionnaire) in twenty-one younger (aged 19–34 years, BMI range: 18–31) and twenty older (aged 60–73 years, BMI range: 19–32) adults matched for BMI. The Alternative Healthy Eating Index was used to quantify diet quality.

**Results:**

Older, compared to younger, individuals reported lower levels of disinhibited eating, consumed a healthier diet, and had weaker connectivity in the frontoparietal (FPN) and default mode (DMN) networks. In addition, associations between functional connectivity and eating behaviour differed between the two age groups. In older adults, disinhibited eating was associated with weaker connectivity in the FPN and DMN––effects that were absent in the younger sample. Importantly, these effects could not be explained by differences in habitual diet.

**Conclusions:**

These findings point to a change in interoceptive signalling as part of the ageing process, which may contribute to behavioural changes in energy intake, and highlight the importance of studying this under researched population.

## Introduction

The problem of obesity affects all age groups but is greatest amongst those aged 60 and over [1]. Increased accumulation of body fat mass, coupled with age-related decline in health, impose a huge burden on health and social care provisions [2]. Therefore, understanding the mechanisms driving obesity in the most vulnerable populations is critical. Neuroimaging offers an insight into the neural underpinnings of obesity. Several neural networks, such as the frontoparietal (FPN) and default mode (DMN) networks, have been associated with obesity and disordered eating [3]. However, studies in older populations are scarce and researchers are yet to compare older and younger populations.

Although older adults are the most at risk of obesity and its complications [4], the desire to lose weight and attempts at dieting are similar across age groups [5]. Interestingly, eating styles that are known to predict obesity, such as disinhibited eating (i.e. the propensity to lose control over food consumption), are less prevalent in older adults, as are hunger sensations [6, 7]. However, the neural mechanisms underlying disordered eating in older adults remain unknown. Recently, functional magnetic resonance imaging (fMRI) based functional connectivity analysis has contributed to our understanding of obesity [8], although most research has considered the connectivity between brain regions in response to specific tasks [9] as opposed to at rest (i.e. resting state). For example, whilst viewing high calorie/palatable foods, obese participants show stronger connectivity between the amygdala, insula, and prefrontal cortex [10]. However, it is also possible to observe functional connectivity during the ‘resting-state’ [11], a task free, intrinsic exploration of interactions between brain regions [12]. This approach bypasses issues concerning food presentation e.g., subjective preference [13]. Interestingly, there are reports that during the resting state those with obesity differ in the connectivity of networks involved with emotional regulation, interoception, self-referential thinking, and inhibitory control [14].

Although most functional connectivity research has been conducted in younger adults, it is notable that activity in the same frontal and parietal networks linked to excess body weight have been found to decline in older individuals [15]. In this context, it is plausible that changes in functional connectivity in older adults might be linked to their differing eating behaviour. For example, it was recently observed that in older adults with obesity, confidence to resist eating was associated with connectivity between attentional control regions of the brain, and the limbic circuitry involved in interoceptive, emotional, and hedonic responses [16]. Although similar effects are reported in young samples [17, 18], researchers have used various scanning/analysis techniques making comparison between ages difficult. In addition, to date no research has directly contrasted older and younger individuals in the same study and under the same experimental conditions. Therefore, we examined the link between resting state functional connectivity and disinhibited eating in younger and older adults who were matched on their BMI, whilst statistically controlling for habitual diet. Specifically, in line with previous research we hypothesised that disinhibited eating would be associated with altered connectivity in the FPN and DMN networks.

## Methods

### Participants

Participants were recruited from the local community using email or poster advertisements. Older adults were recruited from the Dementia Research Group’s volunteer database (Department of Psychology, Swansea University). Participants were excluded if they had implanted magnetic objects/devices or recent tattoos which prohibited entry into the scanning environment, were showing early signs of cognitive decline according to the Montreal Cognitive Assessment [19], were suffering from a clinically relevant metabolic or eating disorder, or were currently dieting or adhering to a specific diet for ethical or other reasons e.g., veganism/vegetarianism. Estimated sample size was based on previous neuroimaging research that had investigated the neural correlates of obesity and eating behaviours [20]. Twenty four younger and 23 older right-handed adults took part after giving written consent. However, during the preprocessing phase, data from six participants (three younger and three older) needed to be removed; one due to anatomical abnormalities and the remainder for technical reasons that made processing impossible, e.g., movement artefacts. Of the remaining sample 21 were younger (aged 19–34, 11 F, 10 M, BMI range 18.4–30.5 kg/m^2^), and 20 were older (aged 60–73, 10 F, 10 M, BMI range 19.4–31.8 kg/m^2^) (Table 1). All participants were naïve to the aims of the study and were required to fast for a period of two hours prior to testing.

**Table 1:** Demographic data of Younger and Older adult groups (mean and standard deviations [SD]).

Participant characteristics	Younger adult group	Older adult group	χ^2^	F ratio	*p* value
Gender	10 M, 11 F	10 M,10 F	0.02	–	.880
Age (years)	23.59 (4.22)	67.01 (3.68)		–	–
Age range	19–34	60–73		–	–
BMI (kg/m^2^)	25.01 (5.12)	26.62 (4.13)		2.63	0.063
DE	19.56 (5.92)	15.0 (4.33)		7.52	0.009*
RE	15.42 (3.84)	17.0 (3.64)		1.60	0.213
EE	6.45 (2.67)	5.95 (2.39)		0.39	0.536
Diet	−3.03 (28.08)	21.13 (30.34)		6.83	0.013*
POMS	32.99 (4.69)	37.02 (3.49)		9.51	0.004*

*N* = 41 (*n* = 21 for younger adult sample and *n* = 20 for older adult sample).

*p < 0.05.

*M* Male, *F* Female, *BMI* Body mass index, *DE* Disinhibited eating, *RE* Restrained eating, *EE* Emotional eating, *POMS* Profile of mood states, Diet Modified healthy eating index.

## Procedure

Upon arrival, participants provided their written informed consent, and height and weight were measured using an electronic weighing scale and a portable stadiometer. They then completed the Profile of Mood States Questionnaire and entered the scanner for a five-minute resting state scan (to improve the reliability of BOLD signal detection and facilitate network delineation, participants’ sustained visual focus on a central cross, placed against a plain background [21]). After the scanning procedure was finished, participants completed the Three Factor Eating Questionnaire and Food Frequency Questionnaire. Ethical approval was gained from the Swansea Psychology Department Ethics Committee and the study was carried out in accordance with the Declaration of Helsinki - Ethical Principles for Medical Research Involving Human Subjects. Derived data supporting the findings of this study are available from the corresponding author on request.

### Three factor eating questionnaire

Eating style was assessed using the Three Factor Eating Questionnaire (TFEQ)-R18 [22, 23]. The TFEQ measures Cognitive Restraint (six items e.g., ‘I deliberately take small helpings as a means of controlling my weight.’), Disinhibited Eating (nine items e.g., ‘Sometimes when I start eating, I just can’t seem to stop’), and Emotional Eating (3 items e.g., ‘When I feel lonely, I console myself by eating.’). Participants were asked to rate their response on a four-item scale ranging from 1 ‘Definitely False’ to 4 ‘Definitely True’. We focused on disinhibited eating, as this dimension most reliably differs according to age and BMI [24]. Importantly, the factor structure of the TFEQ has been replicated across older and younger samples [23]. The internal consistency was moderate for the Cognitive Restraint subscale (Cronbach *α* = 0.67), high for the Disinhibited Eating subscale (Cronbach *α* = 0.89), and high for the Emotional Eating subscale (Cronbach *α* = 0.84).

### EPIC Norfolk food frequency questionnaire

Participants also completed the EPIC Norfolk Food Frequency Questionnaire (FFQ) version 6 - a measure of dietary intake [25]. Participants were asked to rate, on a scale of 0-9 (‘never’ to ‘6+ per day’), the frequency that foods were consumed. A score was calculated for each of the following food groups: fruit, vegetables, ratio of white (e.g., seafood and poultry) to red meat, ratio of polyunsaturated fatty acids to saturated fatty acids, fibre, nuts, and seeds. The sum of these food group scores was then used as a modified version of the Alternate Healthy Eating Index (AHEI) score; a higher score is indicative of a healthier diet [24, 26].

### Profile of mood states

As mood is known to correlate with resting-state functional connectivity [27] and is associated with disinhibited eating [26], there was a need to statistically control for this variable. The Profile of Mood States - Bipolar form [28], is a 72-item Likert-type, self-report measure of mood across 6 dimensions. Six positive and six negative adjectives are listed for each of the six mood dimensions: (1) Composed-Anxious; (2) Energetic-Tired; (3) Elated-Depressed; (4) Clearheaded-Confused; (5) Agreeable-Hostile; (6) Confident-Unsure. Participants were asked to rate each adjective on a four-point scale (1 ‘Much unlike this’ to 4 ‘Much like this’) in order to capture the participants’ mood over the past week. Participants’ overall mood score was calculated by the sum of all six dimensions of mood (following a coding procedure on reversed scoring items). The internal consistency for the POMS-72Q was excellent (Cronbach *α* = 0.83).

### fMRI data acquisition, preprocessing & analysis

fMRI data acquisition and preprocessing have been described elsewhere [29]. Following preprocessing, functional networks were identified for the entire dataset (younger and older adults combined) with group independent component analysis (ICA) using the Group ICA of fMRI Toolbox (GIFT; https://trendscenter.org/software/gift/). ICA is a method of blind source separation, which identifies source signals (independent components) in the fMRI data by maximising the signal’s statistical independence. The resulting Independent Components (ICs) are defined as functional networks in which neural activity operates in concert to generate a statistically independent signal. Each Independent Component (IC) consists of a time-course and a 3D map. The 3D map indicates the spatial extent of the network while the time-course indicates, across time, the strength of the network signal (i.e., functional connectivity) in the data.

Individual images were first normalised by removing the mean value of each image at each time point, and then concatenated across time. After data reduction with principal component analysis, 20 ICs were identified using the infomax algorithm [30]. To estimate the stability of ICs, this analysis was repeated 5 times using ICASSO [31, 32]. Only those ICs with a stability index larger than 0.95 were selected for further analysis. Finally, back reconstruction was applied to estimate the spatial maps and time courses of each IC for each participant using GICA3 [33].

### Overlap of independent components with target networks

The Default Mode (DMN), and Frontoparietal (FPN) networks were chosen as targets because of their established associations with eating behaviour [8, 17, 34, 35]. The auditory network was selected as an additional target network to serve as a control. This allowed us to determine that any associations with the networks of interest were not simply due to more global reductions in functional connectivity, in older versus younger populations [36].

On the group level, the overlap between the 20 ICs with each of the three targets networks was assessed using ICN_atlas (https://icnatlas.com/) [37]. ICN_atlas enables the calculation of 15 metrics of overlap, between an IC and resting-state network templates, defined by a number of atlases [37]. Here, templates from the SMITH10 atlas were chosen because they have been derived from a 20-dimensional ICA and have been matched to behavioural domains using BrainMap [38]. Spatial involvement is defined as the proportion of an intrinsic connectivity network [ICN] that is activated in the input map. In spatial terms, it is the ratio of ICN voxels to ICN volume to capture the degree of engagement of the network in a given activation map [37]. Analysing the spatial properties is often the preferred choice (over temporal properties), given the small number of time points in a given resting-state fMRI dataset [39].

From the set of 20 ICs, the three ICs with the highest spatial involvement measures for the target networks were selected for further analysis. Then, for each participant, the three back-reconstructed ICs identified on the group level were assessed for their overlap with the three target networks using the same procedure. The resulting three overlap measures (spatial involvement) for each participant were used for further analysis.

### Statistical analysis

A multivariate ANOVA was used to detect group differences (Table 1). A chi-squared test was performed on the categorical variable Gender (Table 1). To examine whether the association between resting-state functional connectivity and disinhibited eating varied according to age, a moderated regression analysis was performed using the PROCESS macro (Model 1) for SPSS (v. 25.0, IBM Corp.) version 3.5.2 [40]. A bootstrap sample specified at 5000, and a 95% CI was applied. Resting-state functional connectivity of selected networks (DMN, FPN, and AUD networks) were outcome variables (Y). Disinhibited eating was the predictor variables (X), and age was considered a potential moderator (W) of the (X) and (Y) association. To overcome problems of multicollinearity, variables were mean centred. We conducted separate analyses for each network, where BMI, mood and diet were included as covariates. A False Discovery Rate (FDR) procedure was used to correct the *p*-values of the univariate tests. Statistical significance was set at an *α* = 0.05 with FDR correction [41]. Potential outliers were determined using the Cooks distance diagnostics. To avoid removal of natural variability, we specified a conservative Cook’s distance threshold of 0.2 [42].

## Results

### Demographic/group comparisons

Younger adults (YA) reported significantly higher levels of disinhibited eating (F (1, 39) = 8.61, *p* = 0.006, *η* = 0.987); consumed a poorer quality diet (*F* (1, 39) = 4.82, *p* = 0.034, *η* = 0.110), and reported a less positive mood state (*F* (1, 39) = 9.28, *p* = 0.004, *η* = 0.192) (Table 1). The two samples did not significantly differ on the restraint or emotional eating scale. The groups were well matched on BMI (*F* (1, 39) = 2.62, *p* = 0.113, *η* = 0.063) and gender (χ^2^ (1, *N* = 41) = 0.02, *p* = 0.879, *φ* = 0.02). After the False Discovery Rate correction, mood and disinhibited eating score remained significant.

### Frontoparietal network (FPN)

**Fig. 1:**
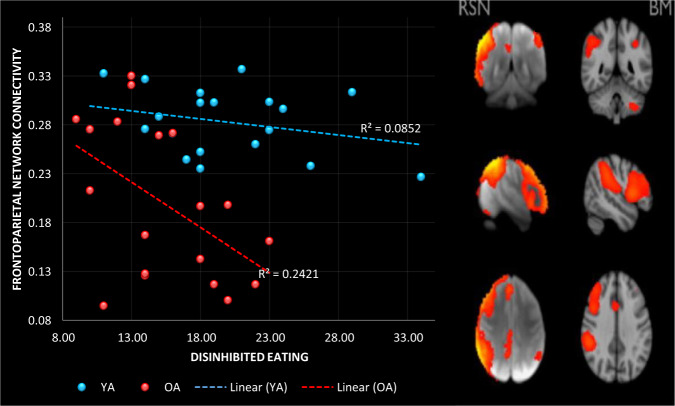
The association between frontoparietal network connectivity and disinhibited eating in older and younger adults. Data do not include the participants removed as identified outliers.

One outlier with a Cook’s distance of 0.29 was removed from the older adult (OA) sample. Overall, the model was significant, accounting for 53% of the variance in FPN connectivity (R^2^ = 0.539, *F* (6, 32) = 6.252, *p* < 0.002; Fig. 1). As expected, OA had weaker connectivity in the FPN (*β* = −0.829, *p* < 0.0001, LLCI −1.154, ULCI −0.504). There was also a significant negative association between DE and FPN connectivity (*β* = −0.431, *p* = 0.003, LLCI −0.713, ULCI −0.148). However, both of these effects were superseded by a significant interaction between DE and age (*β* = −0.402, *p* < 0.013, LLCI −0.714, ULCI −0.091). Probing this interaction revealed that there was a significant negative association between DE and FPN connectivity in OA (*β* = −0.838, *p* < 0.001, LLCI −1.326, ULCI −0.351), but not in YA (*β* = −0.043, *p* = 0.795, LLCI −0.381, ULCI 0.294). Although those with a higher BMI tended to have stronger connectivity in the FPN, the effect was not significant (*β* = −0.272, *p* = 0.056, LLCI −0.008, ULCI 0.551). In addition, neither mood (*β* = −0.059, *p* = 0.662, LLCI −0.332, ULCI 0.214), nor diet quality (*β* = 0.032, *p* = 0.808, LLCI −0.235, ULCI 0.299) were associated with FPN connectivity.

### Default mode network (DMN)

The same participant within the OA sample was removed from this analysis, as he had a Cook’s distance of 0.24. The model was again significant, accounting for 39% of the variance in DMN connectivity (R^2^ = 0.392, *F* (6,32) = 3.433, *p* = 0.010). Similar to the analysis of the FPN, OA had weaker connectivity in the DMN (*β* = −0.708, *p* < 0.001, LLCI −1.082, ULCI −0.334; Fig. S1). However, neither DE (*β* = −0.211, *p* = 0.195, LLCI −0.536, ULCI 0.11 S), BMI (*β* = −0.009, *p* = 0.956, LLCI −0.330, UL 0.313), Mood (*β* = 0.112, *p* = 0.472, LLCI −0.202, UL 0.426) nor Diet (*β* = 0.131, *p* = 0.389, LLCI −0.202, UL 0.438) correlated with DMN connectivity. The interaction between DE and Age (*β* = −0.353, *p* = 0.053, LLCI −0.712, ULCI 0.05) approached significance. Similar to the findings reported above for FPN, this effect reflected a significant negative association between DE and DMN connectivity in OA (*β* = −0.569, *p* = 0.047, LLCI −1.130, ULCI −0.008), but not in YA (*β* = 0.129, *p* = 0.504, LLCI −0.260, ULCI 0.518).

### Auditory network (AUD)

No outliers were identified in the AUD network analysis. Expectedly, the model was not significant (R^2^ = 0.170, *F* (6,33) = 1.124, *p* = 0.370). Likewise, none of the predicted variables were associated with AUD connectivity; Age (*β* = −0.310, *p* = 0.148, LLCI −0.736, UL 0.116), DE (*β* = 0.214, *p* = 0.253, LLCI −0.160, ULCI 0.588), the interaction between DE and age (*β* = −0.012, *p* = 0.950, LLCI −0.408, ULCI 0.384; Fig. S2), BMI (*β* = −0.028, *p* = 0.877, LLCI −0.392, ULCI 0.336), Mood (*β* = 0.152, *p* = 0.404, LLCI −0.214, UL 0.517) and diet (*β* = 0.294, *p* = 0.100, LLCI −0.059, UL 0.647).

## Discussion

As older adults remain a relatively neglected population when examining obesity and eating behaviour, the objective of this study was to determine whether associations between resting state functional connectivity and disordered eating, which have been observed in young adults, are also evident in older adults. The key findings were that: (1) compared to young individuals, older adults reported lower levels of disinhibited eating, and had weaker connectivity in the FPN and DMN, (2) in older adults but not younger adults disinhibited eating was negatively associated with connectivity in both the FPN and DMN (Fig. 1), (3) these effects were not explained by differences in BMI, mood or habitual diet quality, and (4) the specificity of these effects was demonstrated as no associations were observed in the sensitivity analysis of the auditory network.

Despite decades of research into obesity and disordered eating, older samples remain scarcely investigated. Interestingly, obesity rates increase across adulthood [4], however, there is evidence that self-reported disinhibited eating progressively declines between the ages of 40 and 90 years [43]. In addition, a recent meta-analysis reported that healthy older adults (aged 70–74) have 25–39% lower subjective hunger than younger adults (aged 26–27) [7]. The present data support these observations––older individuals reported lower levels of disinhibited eating than those who were younger (Table 1). Notably, over 50% of the items on the TFEQ-18 disinhibited eating scale specifically ask about eating in response to internal cues e.g., “I am always hungry enough to eat at any time”. In studies that have explored the factor analytic structure of eating behaviour, it is not uncommon for ‘hunger’ items to load together with items asking about the ability to control food intake such as “Sometimes when I start eating, I just can’t seem to stop” [44]. Thus responsivity to internal sensations plays a significant role in disinhibited eating, and it is plausible that older adults may report lower levels of disinhibited eating because their experience of hunger is diminished. Future research identifying the processes involved in reduced hunger and disinhibited eating in older populations might be beneficial.

In this context, we observed weaker FPN connectivity in older individuals (Fig. 1). The FPN is an integrated network of domain-general brain regions including frontal, parietal, and anterior insular brain regions that are activated in response to a wide range of task conditions requiring self-regulation and meta-cognition [45]. In addition, the FPN is highly interconnected with other brain networks such as the DMN, and is thought to be involved in their task-related modulation [45]. In particular, there is evidence that a key function of the FPN is to instantiate and flexibly switch self-control in response to feedback [46]. Therefore, the present finding of weaker FPN connectivity in older adults is consistent with evidence that older individuals are generally poorer at information updating, and adapting to changing task demands [47]. Importantly, a body of research has documented that obesity is inversely related to performance on tasks involving working memory, self-control, and cognitive flexibility [48], although fewer studies have considered specific eating styles [49]. Therefore, it is plausible that weaker connectivity among nodes of the FPN involved in flexible self-control may be associated with increased risk of disordered eating. Indeed, the present findings suggest that reduced FPN connectivity may exacerbate disordered eating in older adults (Fig. 1). Future research should explore task-related functional connectivity in relation to cognitive flexibility and eating behaviour in older adults.

In addition, the FPN has significant anterior insular and dorsal ACC connections (Fig. 1). These areas of the brain are often associated with the prediction, detection and filtering of salient afferent signals, especially those originating from inside the body (interoceptive signals) [50, 51]. This suggests that the FPN may also have a role in regulating the internal state by guiding relevant behaviours that generate the expected interoceptive inputs [51]. In this way reduced connectivity in the FPN in older adults, may interfere with the anticipation and regulation of the energy needs of the body, contributing to dysregulated styles of eating.

Consistent with prior research [52], we also observed that older adults had weaker connectivity within the DMN, which comprises the medial parietal (precuneus and posterior cingulate), hippocampus, and prefrontal cortices. In addition, in older adults weaker DMN connectivity correlated negatively with disinhibited eating, meaning that weaker DMN connectivity is demonstrated particularly in those older adults with high levels of disinhibited eating. Research indicates that the DMN may be a key network involved in the representation of the brains internal model of the world [50, 51]. This internal model is used to inform predictions about sensory inputs, including predictions about ongoing changes to the body’s internal milieu [50, 51]. In the context of eating behaviour weaker DMN connectivity might reflect a diminished capacity of this network to categorise and integrate afferent information with internally generated concepts [14, 51]. Although speculative, this interpretation would be consistent with evidence suggesting that older adults’ mental representations of emotion are less associated with interoceptive sensations than are those of younger adults [53], and that poorly differentiated emotional and interoceptive experiences might exacerbate disordered eating [54]. This suggests that future studies assessing the role of emotion differentiation might be profitable. For instance, here we chose to control for mood, however, as older adults reported a better mood (Table 1), and a poorer mood was previously linked to disinhibited eating [26], future research might profit from considering the role of mood and / or emotion in age-related declines in disinhibited eating, and whether any such effects are related to network connectivity.

Interestingly, the present observation that FPN connectivity was not associated with disinhibited eating in younger adults (Fig. 1) contrasts with previous findings [3]. Park et al. (2016) examined functional connectivity in healthy weight (aged 29.83 (9.95)) and overweight (aged 33.24 (10.09)) individuals, and observed positive associations between the FPN, BMI, and disinhibited eating [3]. One explanation for our inability to replicate this effect is our fairly small sample size, although our sample size was sufficient to observe a significant negative association in the older group (Fig. 1). A second explanation is that here we controlled for a number of important confounds which have not been considered in prior research [3]. For example, our samples were matched on BMI, and we controlled for differences in mood and habitual diet quality. Mood and diet were previously found to influence brain functioning [14, 27], and in the present study varied according to age (Table 1). Regardless of the explanation, the present data are important as they suggest that findings obtained from young undergraduate populations cannot be assumed to translate to older populations, who are most at risk from obesity and its comorbidities. In the future, better characterisation of research samples will be needed to understand phenotypic differences, and their association to the activation patterns of neural networks and to personalise interventions.

A strength of the current study is that we were able to demonstrate the specificity of our findings to the FPN and DMN. The sensitivity analysis showed no associations between disinhibited eating, age and connectivity in the auditory network (Fig. S2). The auditory network includes the primary and association auditory cortices, superior temporal gyrus, Heschl’s gyrus, and notably the posterior insular cortex. It was proposed that in the insular cortex there is a posterior-to-anterior gradient [55], with the posterior insula processing the physical features of interoception, while the anterior insula being responsible for the integration of interoception with cognitive and motivational information, and the subjective awareness of feelings [56]. Taking this view, the present findings suggested that older adults with disinhibited eating may have particular problems integrating afferent sensations with higher order cognitive and affective processes. Future research focusing on functional connectivity in insular sub-regions will be needed to determine if this is the case. Interestingly, it was previously observed that age related declines in connectivity were more apparent in the dorsal insula (associated with executive functioning), while connectivity in the ventral insula (associated with affect) is relatively spared [57]. Therefore, future research scrutinising functional connectivity in insular sub-regions might be profitable.

Due to the cross-sectional design of this research reverse causality cannot be ruled out. For instance, an alternative explanation for the present results is that a lifetime of disinhibited eating combined with the aging process (which is known to affect cerebral vascularisation) results in weaker FPN and DMN connectivity. However, if this were the case we would expect habitual diet quality and / or BMI to have influenced the results and they did not. Nonetheless, longitudinal data will be required to fully exclude this possibility. A second limitation relates to our small sample size: it is possible that we lacked the power to detect significant effects in our younger sample. It should also be considered that when comparing cross-sectional groups (e.g., younger vs. older adults) small sample sizes can increase the likelihood of the groups being confounded by unmeasured individual differences. Although we controlled for known differences (i.e., mood, habitual diet, BMI), it is possible that unknown individual differences remain. Therefore, future replication of these findings within larger sample sizes is crucial. Thirdly, although our choice of target networks was theoretically driven, the observed effects may extend beyond those networks. For example, a wider network of brain regions (bilateral frontal and parietal regions, amygdala, temporal pole, hippocampus, fusiform gyrus, and inferior insula) were previously associated with confidence in resisting eating in the absence of hunger [16]. Future research comparing older and younger adults might profit from assessing global brain connectivity and / or applying graph theoretic metrics that are able to provide a more comprehensive understanding of the network topology and their interactions. Finally, we used the Profile of Mood States bipolar scale which prevents us from examining the individual effects of positive and negative affect. Future research might explore the potential differential contributions of positive and negative affect.

In conclusion, we observed age-related associations between disinhibited eating and resting state connectivity in the frontoparietal and default mode networks. These observations may point to a change in interoceptive signalling which contribute to behavioural changes in energy intake during senescence.

## Supplementary information

Supplementary info
