# Supplementary material for: Weaker connectivity in resting state networks is associated with disinhibited eating in older adults
Source: Int J Obes (Lond). 2022 Jan 11;46(4):859–65. doi: 10.1038/s41366-021-01056-1 (PMC8960408; doi:10.1038/s41366-021-01056-1)
Supplement: Supplementary file 1 — Supplementary info [file 41366_2021_1056_MOESM1_ESM.docx]

**Weaker Connectivity in Resting State Networks is Associated with Disinhibited Eating in Older Adults.**

Anthony Brennan, Lars Marstaller, Hana Burianová, David Benton,

Claire J. Hanley, Simon Newstead and Hayley A. Young.

Supplementary information

**Figure S1. *The association between default mode network connectivity and disinhibited eating in older and younger adults***

*Note:* Data do not include the participants removed as identified outliers.

**Figure S2. *The association between auditory network connectivity and disinhibited eating in older and younger adults***

*Note:* Data do not include the participants removed as identified outliers.

**The Three-Factor Eating Questionnaire**

Please read each statement and select from the multiple choice options the answer that indicates the frequency with which you find yourself feeling or experiencing what is being described in the statements below.

1. When I smell a delicious food, I find it very difficult to keep from eating, even if I have just finished a meal.

Definitely true (4)/ mostly true (3)/ mostly false (2)/ definitely false (1)

2. I deliberately take small helpings as a means of controlling my weight.

Definitely true (4)/ mostly true (3)/ mostly false (2)/ definitely false (1)

3. When I feel anxious, I find myself eating.

Definitely true (4)/ mostly true (3)/ mostly false (2)/ definitely false (1)

4. Sometimes when I start eating, I just can’t seem to stop.

Definitely true (4)/ mostly true (3)/ mostly false (2)/ definitely false (1)

5. Being with someone who is eating often makes me hungry enough to eat also.

Definitely true (4)/ mostly true (3)/ mostly false (2)/ definitely false (1)

6. When I feel blue, I often overeat.

Definitely true (4)/ mostly true (3)/ mostly false (2)/ definitely false (1)

7. When I see a real delicacy, I often get so hungry that I have to eat right away.

Definitely true (4)/ mostly true (3)/ mostly false (2)/ definitely false (1)

8. I get so hungry that my stomach often seems like a bottomless pit.

Definitely true (4)/ mostly true (3)/ mostly false (2)/ definitely false (1)

9. I am always hungry so it is hard for me to stop eating before I finish the food on my plate.

Definitely true (4)/ mostly true (3)/ mostly false (2)/ definitely false (1)

10. When I feel lonely, I console myself by eating.

Definitely true (4)/ mostly true (3)/ mostly false (2)/ definitely false (1)

11. I consciously hold back at meals in order not to weight gain.

Definitely true (4)/ mostly true (3)/ mostly false (2)/ definitely false (1)

12. I do not eat some foods because they make me fat.

Definitely true (4)/ mostly true (3)/ mostly false (2)/ definitely false (1)

13. I am always hungry enough to eat at any time.

Definitely true (4)/ mostly true (3)/ mostly false (2)/ definitely false (1)

14. How often do you feel hungry?

Only at meal times (1)/ sometimes between meals (2)/ often between meals (3)/almost always (4)

15. How frequently do you avoid “stocking up” on tempting foods?

Almost never (1)/ seldom (2)/ moderately likely (3)/ almost always (4)

16. How likely are you to consciously eat less than you want?

Unlikely (1)/ slightly likely (2)/ moderately likely (3)/ very likely (4)

17. Do you go on eating binges though you are not hungry?

Never (1)/ rarely (2)/ sometimes (3)/ at least once a week (4)

18. On a scale of 1 to 8, where 1 means no restraint in eating (eating whatever you want, whenever you want it ) and 8 means total restraint (constantly limiting food intake and never “giving in”), what number would you give yourself?
